# Multiple bloodmeals enhance dissemination of arboviruses in three medically relevant mosquito genera

**DOI:** 10.1186/s13071-024-06531-y

**Published:** 2024-10-19

**Authors:** Zannatul Ferdous, Constentin Dieme, Hannah Sproch, Laura D. Kramer, Alexander T. Ciota, Doug E. Brackney, Philip M. Armstrong

**Affiliations:** 1 Department of Entomology, The Center for Vector Biology and Zoonotic Diseases, The Connecticut Agricultural Experiment Station, 123 Huntington St., New Haven, CT 06511 USA; 2 The Arbovirus Laboratory, Wadsworth Center, New York State Department of Health, Slingerlands, NY USA

## Abstract

**Background:**

Mosquitoes in nature may acquire multiple bloodmeals (BMs) over the course of their lifetime; however, incorporation of frequent feeding behavior in laboratory vector competence studies is rarely done. We have previously shown that acquisition of a second non-infectious BM can enhance early dissemination of Zika virus (ZIKV), dengue virus, and chikungunya virus in *Aedes aegypti* and ZIKV in *Aedes albopictus* mosquitoes, yet it is unknown if other taxonomically-diverse virus-vector pairings show a similar trend under a sequential feeding regimen.

**Methods:**

To test this, we evaluated the impact of a second noninfectious BM on the vector competence of *Aedes aegypti* and *Anopheles quadrimaculatus* for Mayaro virus, *Culex quinquefasciatus* for West Nile virus, *Aedes triseriatus* for La Crosse virus, and *Aedes aegypti* for Oropouche virus (OROV). Female mosquitoes were fed BMs containing these viruses and half of them were given a second noninfectious BM at 3 or 4-days post infection. Mosquitoes were harvested at various time points and assayed for virus infection in bodies and disseminated infection in legs by performing reverse transcription-quantitative polymerase chain reaction (RT-qPCR) assays.

**Results:**

We found that a second noninfectious BM had no impact on midgut infection rates but increased virus dissemination for all but one of the virus-vector pairings- *Ae*. *aegypti* and OROV. Unlike the other arboviruses under consideration, which are strictly mosquito-borne, biting midges (*Culicoides* spp.) serve as the main vector of OROV and this virus rarely disseminated to the mosquito leg tissue in our study.

**Conclusions:**

Taken together, our findings show that sequential blood feeding enhances virus dissemination across diverse arbovirus-vector pairings, representing three mosquito genera and virus families, but a second BM was insufficient to overcome a strong midgut virus escape barrier in a nonnatural virus–vector pairing.

**Graphical Abstract:**

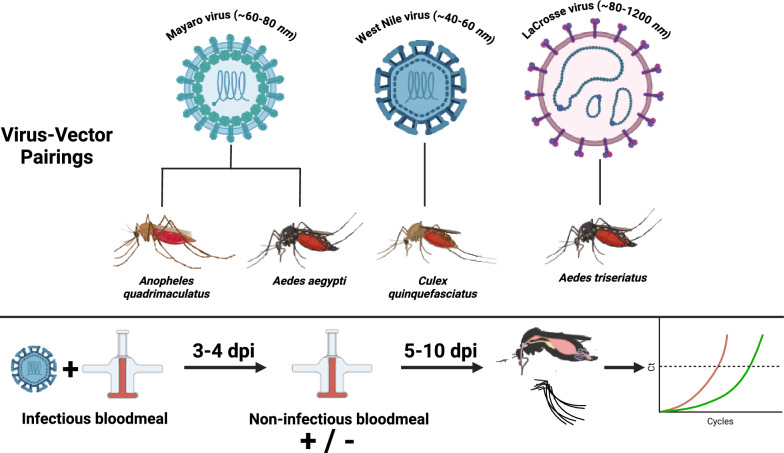

## Background

Arthropod-borne viruses (arboviruses) continue to pose a significant threat to public health worldwide. This is highlighted by the sustained transmission of dengue virus (DENV; *Flavivirus*, *Flaviviridae*) throughout the tropics [1, 2], the emergent epidemics of West Nile virus (WNV; *Flavivirus*, *Flaviviridae*) [1, 3], Zika virus (ZIKV; *Flavivirus*, *Flaviviridae*) [1, 2], Mayaro virus (MAYV; *Alphavirus*, *Togaviridae*) [4], and Oropouche virus (OROV; *Orthobunyavirus*, *Peribunyaviridae*) [5] in the Americas, and recurrent cases of La Crosse virus (LACV; *Orthobunyavirus*, *Peribunyaviridae*) encephalitis in eastern North America [6]. Vaccines and therapeutics are limited or not available for these and other arboviruses. Therefore, the most effective interventions rely on mosquito control and other prevention measures that require an in-depth understanding of the vector biology and epidemic risk of arboviruses.

To better understand the dynamics of arbovirus transmission and vulnerabilities within the transmission cycle, we need to examine the assumptions underlying virus–vector–host interactions. For example, both vector competence (the ability of a vector to become infected and transmit a pathogen) and extrinsic incubation period (EIP; duration from pathogen acquisition to transmission by the vector) are empirically determined by offering mosquitoes an infectious bloodmeal (BM) and periodically sampling mosquitoes at different time points post infection to determine infection and transmission status [7]. This approach is the gold standard and has been used for decades; however, mosquitoes in nature may imbibe several BMs over the course of the EIP, with higher feeding frequency for some species, such as *Aedes aegypti* and *Anopheles gambiae*, that can take multiple BMs per gonotrophic cycle [8]. Previously, we found that providing a second noninfectious BM to ZIKV infected *Ae*. *aegypti* and *Ae*. *albopictus* mosquitoes enhances virus escape from the midgut and significantly shortens the duration of the EIP [9, 10]. We also showed that additional noninfectious meals increased DENV and Chikungunya virus (CHIKV) escape from *Ae*. *aegypti*’s midgut. These findings suggest that current protocols assessing the competency of mosquito populations may underestimate the potential risk associated with mosquito-borne viruses; nevertheless, it is unclear whether this double feed phenomenon is more universal to other arbovirus-vector systems.

We previously found that mosquito midgut expansion during blood feeding temporarily compromises the integrity of the surrounding basal lamina layer, thereby providing a possible mechanism for enhanced virus escape during a second BM [9–12]. If this mechanism is correct, then we expect that multiple blood feeding episodes will significantly enhance virus dissemination for taxonomically diverse arbovirus–vector pairings. To address this possibility, we evaluated the impact of a second noninfectious BM on the vector competence of *Ae*. *aegypti* for MAYV and OROV, *Anopheles quadrimaculatus* for MAYV, *Culex quinquefasciatus* for WNV and *Ae*. *triseriatus* for LACV. Mosquito species were exposed to infectious BMs with each of the viruses and 3–4 days post infection (dpi) a second noninfectious BM was offered to half of the infected individuals. Subsequently, mosquito infection and dissemination status were determined by reverse transcription-quantitative polymerase chain reaction (RT-qPCR) at multiple time points post infection for single- and double-feed groups [13].

## Methods

### Viruses, cell culture, mosquitoes

Viruses from three different families were used in this study: *Togaviridae* (MAYV BeH256, GenBank KP842819), *Flaviviridae* (WNV 2741-99, GenBank AF206518), and *Peribunyaviridae* (LACV, 78 V-8853, GenBank MT276617; OROV BeH472200, GenBank AF164537). Virus stocks were prepared by amplifying them in C6/36 cells (WNV and OROV) or Vero cells (MAYV and LACV). Cells were maintained in Minimal Essential Medium (MEM) with 10% fetal bovine serum, 100 U/ml penicillin, 100 mg/ml streptomycin, D-glutamine, 50 mg/ml amphotericin B, and sodium bicarbonate at 28 °C, 5% CO_2_ for C6/36 cells or 37 °C, 5% CO_2_ for Vero cells. We used frozen stocks for vector competence studies for all arboviruses except WNV, which were grown fresh prior to each experiment [14]. Mosquito colonies used in this study were *Ae*. *aegypti* (Orlando strain, collected in 1952 from Orlando, FL), *Ae*. *quadrimaculatus* (Orlando strain, MRA-139, BEI Resources, Manassas, VA), *Cx*. *quinquefasciatus* (Benzon Research Inc., Carlisle, PA) and *Ae*. *triseriatus* (collected in Waterford, CT in 1992). Mosquitoes were maintained in an insectary at 27 °C, 70% relative humidity, and a 14:10 h light–dark cycle. Larvae were reared in pans of water and fed a 2% solution of 3:2 liver powder and brewer’s yeast mix. Adults were housed in 30 × 30 × 30 cm cages and provided a 10% sucrose solution on soaked cotton balls.

### Vector competence study

A mixture of virus and defibrinated sheep blood was offered to 5–7 day-old female mosquitoes. Before blood feeding, mosquitoes were sugar starved for 24 h. Mosquitoes were offered infectious BMs using a glass water-jacketed membrane feeder connected to a circulating 37 °C water bath with an intestine sausage casing as a membrane. Final BM titers are as follows MAYV: 1.0 × 10^6^ PFU/mL, LACV: 7.0 X 10^5^ PFU/mL, OROV: 1.0 X 10^6^ PFU/mL, and WNV: 3.6–9.7 X 10^5^ PFU/mL. After feeding for an hour, mosquitoes were cold-anesthetized to transfer fully engorged females into two 32-oz ice cream cartons. Each carton was housed in an environmental chamber at 28 °C 14:10 h light–dark cycle with an egg cup and egg-laying paper. During incubation, mosquitoes received 10% sugar solution.

Half of the mosquitoes were offered a second BM (no virus added) 3 days postinfection (DPI) for *Ae*. *aegypti* and *Ae*. *quadrimaculatus* and 4 DPI for *Ae*. *triseriatus* and *Cx*. *quinquefasciatus*. After being cold anesthetized, fully engorged females were placed in a new carton with egg laying cups and provided with 10% sucrose solution. Mosquitoes were harvested at the end of the incubation period and legs were removed using flame-sterilized forceps. Mosquito bodies and legs were homogenized separately in 250 ul phosphate-buffered saline with 0.5% gelatin, 30% heat-inactivated rabbit serum, 1 × antibiotic–antimycotic (PBS-G) in a 2 mL microcentrifuge tube with a copper ball bearing (BB) and using a mixer mill set at 24 cycles/s for 30–60 s.

### RT-qPCR for detecting viral RNA

Total RNA was extracted from 50 μl of mosquito leg and body homogenates using the Mag-Bind Viral DNA/RNA 96 Kit (Omega Bio-tek Inc., Norcross, GA) on a Kingfisher Flex automated nucleic acid extraction device (ThermoFisher Scientific, Waltham, MA) following the manufacturer’s instructions. Samples were eluted in 50 μl ddH2O and screened for viral RNA using previously described primer–probe sets for WNV, MAYV, OROV, and LACV [15–17]. The same RT-qPCR protocol was used to detect all four viruses. In brief, 25 μl reactions containing 2.5 μl of total RNA were assayed with the TaqMan RNA-to-Ct 1-Step Kit (ThermoFisher Scientific) on a CFX96 Touch Real-Time PCR Detection System (Bio-Rad, Hercules, CA) using the following parameters: RT—50 °C for 30 min, 95 °C for 10 min, PCR—95 °C for 15 s., 60 °C for 1 min followed by a plate read (50 cycles). Data were analyzed using the Bio-Rad CFX Manager 3.1 software. The cycle threshold (Ct) value to be considered positive by RT-qPCR was < 37 for MAYV and OROV, < 36 cycles for LACV, and < 35 cycles for WNV.

### Data analysis

Fisher’s exact test was used to analyze differences in the proportion of mosquitoes that had midgut and disseminated infections at each time point. In addition, we evaluated overall differences among groups at multiple time points by analysis of covariance (ANCOVA). Linear regression lines were fitted over time and evaluated for differences in slope and y intercept. The standard error of the sample proportions was used to calculate the error bars. Each fig. legend provides descriptive statistics. GraphPad Prism Statistical software was used for all analyses.

## Results

**Fig. 1:**
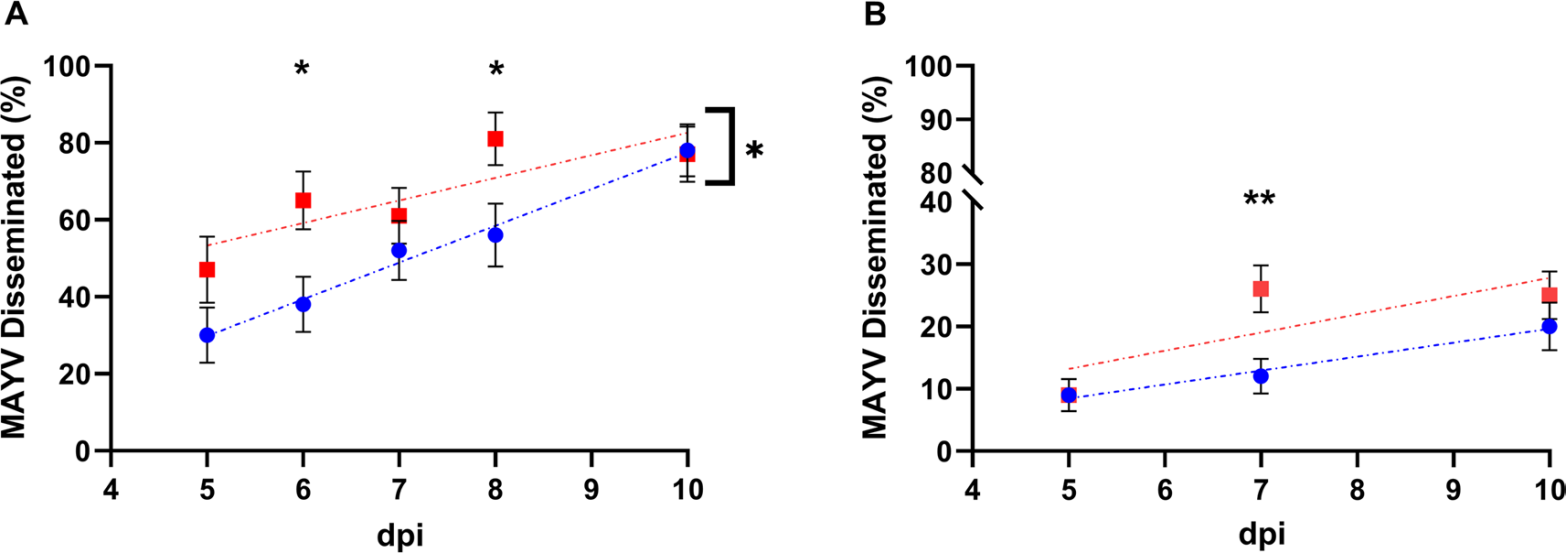
Comparison of Mayaro virus disseminated infection rates in single-fed (blue) and double-fed (red) mosquitoes for **A** *Ae*. *aegypti* and **B** *Ae*. *quadrimaculatus*. The data for each day post-infection (dpi) were analyzed by a two-sided Fisher’s exact test. Data were also fitted into linear regression lines across all time points and compared by analysis of covariance indicated by brackets. *P < 0.05; **P < 0.01; ***P < 0.001. Error bars represent the binomial stand error of the mean of sample proportions

To determine whether multiple BMs enhance dissemination of an *Alphavirus* in two evolutionarily distinct mosquito species, *Ae*. *aegypti* and *Ae*. *quadrimaculatus* mosquitoes were orally exposed to MAYV followed by a second noninfectious BM to the double feed group (DFG) at 3 DPI. Infection status was compared with those receiving only an infectious BM- single feed group (SFG) at 5–10 DPI. MAYV infection rates ranged from 50% to 80% and were not significantly different between the SFG and DFG for both mosquito species at all time points (Table 1). In contrast, the percentage of mosquitoes with disseminated infection was significantly higher in the DFG than the SFG at days 6 and 8 DPI (Fisher’s exact test, *P* < 0.05) for *Ae*. *aegypti* but this difference in dissemination disappeared by day 10 (Table 1, Fig. 1A). Similarly, *Ae*. *quadrimaculatus* fed a second BM had higher rates of disseminated infection than those fed once at day 7 DPI (Fisher exact test, *P* < 0.01) (Fig. 1B). The overall trend indicated higher rates of disseminated infection in the DFG for both mosquito species, but these differences were not statistically significant at all time points. Therefore, we reanalyzed the data by ANCOVA to provide a summary statistic for viral disseminated infection rates across the time series. There were no significant differences in the slope of regression lines for the SFG and DFG, but the y-intercept was significantly higher for the DFG for *Ae*. *aegypti* (ANCOVA, *P* < 0.05; Fig. 1A) indicating overall higher rates of dissemination in these mosquitoes.

**Table 1:** Summary of experimental data evaluating the impact of a second noninfectious blood meal on the infection and dissemination rates of Mayaro virus for *Aedes aegypti* and *Anopheles quadrimaculatus*, West Nile virus for *Culex quinquefasciatus*, La Crosse virus for *Aedes triseriatus*, and Oropouche virus for *Aedes aegypti*

Mosquito species	Virus	DPI^a^	Feeding status^b^	MGI (%)^c^	DI (%)^d^
*Ae*. *aegypti*	MAYV	5	SF	40/65 (62)	12/40 (30)
			DF	34/65 (52)	16/34 (47)
		6	SF	44/66 (67)	17/44 (38)
			DF	40/66 (61)	26/40 (65)
		7	SF	42/64 (66)	22/42 (52)
			DF	44/62 (71)	27/44 (61)
		8	SF	36/65 (55)	20/36 (56)
			DF	32/64 (50)	26/32 (81)
		10	SF	37/53 (70)	29/37 (78)
			DF	34/54 (63)	26/34 (77)
*Ae*. *quadrimaculatus*	MAYV	5	SF	121/180 (67)	11/121 (9)
			DF	129/180 (72)	12/132 (9)
		7	SF	128/160 (80)	15/128 (12)
			DF	131/180 (73)	34/131 (26)
		10	DF	108/172 (63)	22/108 (20)
			DF	127/180 (71)	32/127 (25)
*Cx*. *quinquefasciatus*	WNV	6	SF	59/68 (87)	13/59 (22)
			DF	59/64 (92)	23/59 (39)
		8	SF	62/68 (91)	25/62 (40)
			DF	37/45 (82)	26/37 (70)
		10	SF	62/68 (91)	27/62 (44)
			DF	47/53 (89)	33/47 (70)
*Ae*. *triseriatus*	LACV	6	SF	98/107 (92)	46/98 (47)
			DF	78/91 (84)	51/78 (65)
		8	SF	80/102 (78)	58/80 (73)
			DF	62/92 (67)	57/62 (92)
*Ae*. *aegypti*	OROV	7	SF	20/67 (30)	1/20 (5)
			DF	25/65 (39)	1/25 (4)
		14	SF	17/65 (26)	0/17 (0)
			DF	18/64 (28)	1/18 (6)

^a^*DPI* days post infection

^b^*SF* single feed; DF = double feed

^c^*MGI* midgut infection

^d^*DI* disseminated infection

**Fig. 2:**
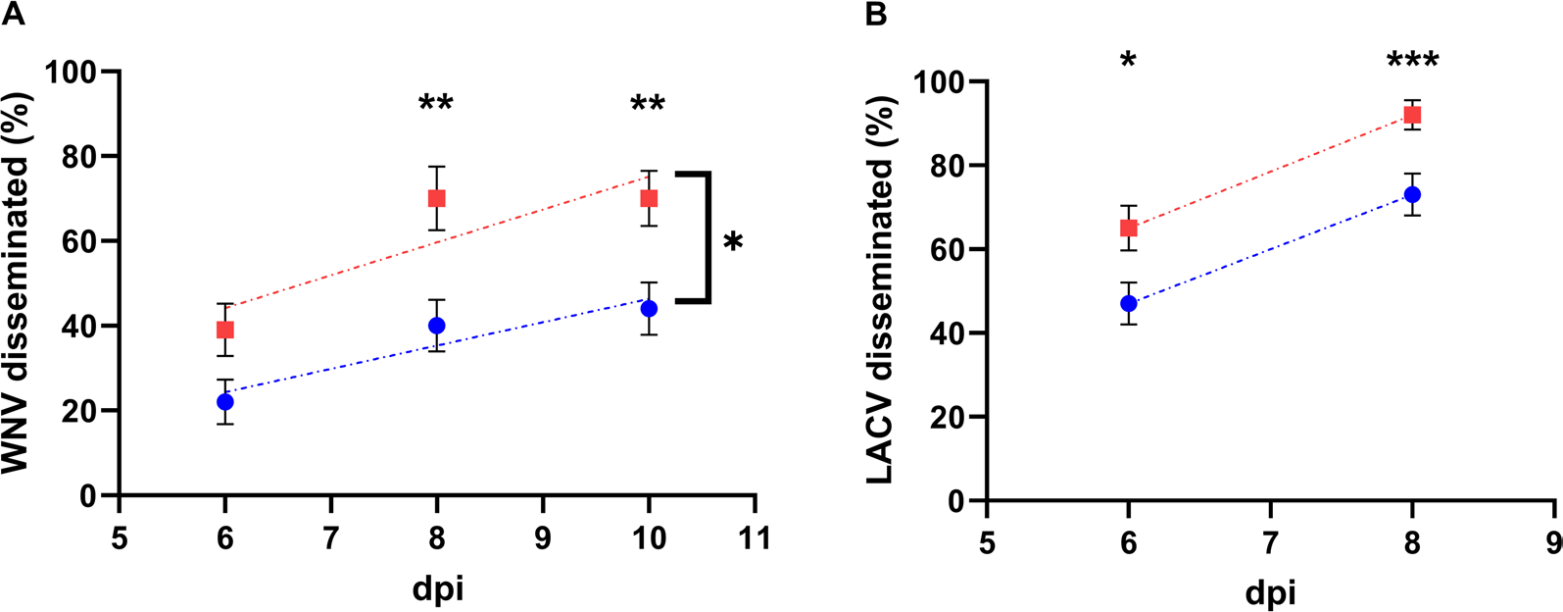
Comparison of virus disseminated infection rates in single-fed (blue) and double-fed (red) mosquitoes for **A** West Nile virus and *Cx*. *quinquefasciatus* and **B** La Crosse virus and *Ae*. *triseriatus*. The data for each day postinfection (dpi) were analyzed by a two-sided Fisher’s exact test. Data were also fitted into linear regression lines across all time points and compared by analysis of covariance indicated by brackets. *P < 0.05; **P < 0.01; ***P < 0.001. Error bars represent the binomial stand error of the mean of sample proportions

We then examined the impact of a second non-infectious BM on *Flavivirus*-Culex interactions by testing WNV dissemination in *Cx*. *quinquefasciatus*. Midgut infection varied from 82% to 92% and were statistically similar between the SFG and DFG across all time points (Table 1). Virus dissemination rates, in contrast, increased over time and were higher for the DFG at 8 and 10 DPI (Fisher Exact Test, *P* < 0.01) (Table 1, Fig. 2A). Significant differences in virus dissemination were also detected by comparing the y-intercept of SFG and DFG regression lines (ANCOVA, *P* < 0.05).

Finally, we evaluated the effects of an additional BM on *Orthobunyavirus* dissemination by testing *Ae*. *triseriatus*-LACV and *Ae*. *aegypti*-OROV pairings. Midgut infection prevalence was similar among SF and DF groups for *Ae*. *triseriatus* and LACV; however, disseminated infection rates were significantly higher for the DFG at both time points (Fisher exact test, 6 DPI *P* < 0.05, 8 DPI *P* < 0.001) (Table 1, Fig. 2B). These results could not be analyzed by ANCOVA with data from only two time points. In contrast, a second noninfectious BM did not increase OROV dissemination in *Ae*. *aegypti*. Only 26–39% of these mosquitoes became infected and 0–6% developed disseminated infection with no significant differences among groups (Table 1).

## Discussion

In this study, we show that additional noninfectious BMs promote virus dissemination across diverse virus-vector pairings, representing three mosquito genera (*Aedes*, *Anopheles*, and *Culex*) and three virus families (*Flavivirdae*, *Orthobunyaviridae*, and *Togaviridae*). These findings reinforce results from an earlier study demonstrating that a second BM enhanced virus midgut escape of CHIKV, DENV, and ZIKV in *Ae*. *aegypti* and ZIKV in *Ae*. *albopictus*, indicating that this phenomenon is broadly applicable across many virus-mosquito systems [9]. The only exception was OROV, which is transmitted by biting midges with *Culicoides paraensis* serving as the main vector in the urban transmission cycle [18]. Although this virus has also been isolated from mosquitoes during epidemics, their role in supporting OROV transmission remains unclear [19]. We found that about a third of *Ae*. *aegypti* acquired OROV infection but they rarely developed disseminated infections, and that a second BM was insufficient to overcome this barrier to virus spread within the vector. Our findings agree with another vector competence study showing that *Ae*. *aegypti* are poor vectors of OROV; however, in that study mosquitoes were completely refractory to midgut infection [20]. Together, our findings indicate that a second BM enhances virus dissemination within competent vectors but failed to boost the competency of a non-natural virus–vector pairing with a strong midgut escape barrier.

The results of the double feed experiments provide support for a common mechanism for virus dissemination in multiple virus–vector systems. After the virus has established infection within the mosquito midgut, it must traverse the surrounding basal lamina layer to gain access to the hemocoel and disseminate to peripheral tissues, including the salivary glands [7]. The basal lamina has a pore size of ~ 9–11 nm, yet arboviruses with larger diameters (40–110 nm) can circumvent this barrier [10, 21]. We and others have previously shown that the integrity of the basal lamina layer is temporarily degraded after a BM, which provides a possible mechanism for virus midgut escape [9, 11, 12, 22, 23]. Specifically, basal lamina damage, measured by monitoring collagen IV damage, spiked immediately after blood feeding and remained elevated for 36 h postfeeding, and microperforations formed in the basal lamina layer that could serve as conduits for virus dissemination. In other experiments, mosquitoes were infected with CHIKV or ZIKV by intrathoracic inoculation and half were given a non-infectious BM 1–3 days post inoculation [9, 22]. Midgut epithelial cells did not become infected unless mosquitoes were given a BM after inoculation, indicating that the basal lamina barrier becomes permissive to virus dissemination only after blood feeding. Our current working model is that midgut expansion during blood feeding induces structural damage to the integrity of the basal lamina layer, which makes it more porous [9]. The basal lamina undergoes repair after blood feeding but never returns to the unfed state which allows for continued virus escape from the midgut or possibly, there is some baseline level of basal lamina leakiness prior to a BM [11]. Regardless, a second noninfectious BM increases the amount of damage to the basal lamina when the virus is already seeded in the midgut thereby increasing the likelihood of virus escape [10]. If this model is correct, then a second BM should cause biophysical changes to the basal lamina that will enhance dissemination in diverse virus–mosquito systems. This was observed in this study in all but one virus–vector pairing (OROV-*Ae*. *aegypti*).

It is unclear why OROV was unable to effectively disseminate within *Ae*. *aegypti* even when given a second BM. One possibility is that the virus poorly infected the midgut epithelium. Arboviruses typically infect a few cells during initial midgut infection but then virus foci expand across the midgut epithelium by spreading to neighboring cells [24–26]. If the virus foci fail to expand within the midgut, then the likelihood that virus-infected cells overlap with breaks or weaknesses in the basal lamina layer decreases. Nevertheless, if this scenario is true, then virus dissemination should still increase when mosquitoes are given a second BM by increasing the number of blood-meal induced microperforations in the basal lamina layer [9]. Another possibility is that OROV escaped from the midgut but was unable to establish infection and replicate within the peripheral tissues. Few virus particles are expected to successfully traverse the midgut basal lamina into the surrounding hemolymph and they could be vulnerable to antiviral defenses of the immune system [27]. Given that mosquitoes are not the natural host of OROV, this virus may be maladapted for replication within this hostile environment. In follow-up experiments, we infected *Ae*. *aegypti* with OROV by intrathoracic inoculation of 17.3 PFU of virus (data not shown), indicating that this mosquito can support disseminated infection after circumventing the midgut escape barrier. These findings were supported by another study showing that OROV can readily replicate within the mosquito after intrathoracic inoculation [20]. Perhaps the midgut escape barrier exerts a powerful bottleneck on the virus population and an insufficient number of viruses escape the midgut to establish infection in peripheral tissues.

## Conclusions

Our findings further emphasize the importance of multiple mosquito BMs on virus dissemination and vector competence that has potential implication for virus transmission in the field. Existing vector competence studies using a single BM exposure may underestimate vector competence particularly in species that take frequent BMs, such as *Ae*. *aegypti* [8]. We previously modeled the impact of a second BM on the transmission of ZIKV by *Ae*. *aegypti* and found that it resulted in a significant increase in the basic reproductive number of the pathogen [9]. This could help explain how *Ae*. *aegypti* could sustain explosive ZIKV epidemics despite its low vector competence in single feed experiments. This study shows that this phenomenon is more broadly applicable to other virus–vector systems and that the feeding behavior of mosquito species should be considered when performing vector competence trials.

## Data Availability

All data generated or analyzed during this study are included in this published article.
